# Study on mechanical properties and acoustic emission characteristics of deep diorite under uniaxial compression

**DOI:** 10.1016/j.heliyon.2024.e24482

**Published:** 2024-01-11

**Authors:** Kai-De Liu, Yu Zhou, Xiao-Ping Zhang, Shao-Jun Fu, Quan-Sheng Liu, Peng Dong, Kai-Wen Yao, Ding-Bo Wang

**Affiliations:** a Xijing University, Shaanxi Key Laboratory of Safety and Durability of Concrete Structures, Xi'an, 710123, China; b The Key Laboratory of Safety for Geotechnical and Structural Engineering of Hubei Provine, School of Civil Engineering, Wuhan University, Wuhan, 430072, China; c Hanjiang –to –Weihe River Valley Water Diversion Project Construction Co., Let., Xi’ an, 710024, China

**Keywords:** Mechanical properties of uniaxial compression, Acoustic emission characteristics, Ringing count, Energy, RA-AF, Anchor point, Damage mechanism

## Abstract

The research object is diorite in the Lingbei TBM section of the Hanjiang-To-Weihe River Qinling tunnel, with a buried depth of over 1 km. Using MTS-2000 microcomputer-controlled electro-hydraulic servo universal testing machine and DS5-16b acoustic emission (AE) monitoring system, uniaxial compression and acoustic emission monitoring tests were carried out on rock samples, to study the uniaxial compression mechanical properties and acoustic emission characteristics of the deep diorite. The results of the study indicate that: (1) During uniaxial compression, diorite undergoes four stages: initial compaction, elasticity, yield and failure, in which the curve of the initial compaction stage is significantly smoother. The uniaxial compressive strength is 41.95 MPã102.42 MPa, with an average of 74.07 Mpa; The axial peak strain ranges from 1 % to 1.4 %, and the failure mode belongs to brittle ductile splitting failure. (2) The cumulative ringing count and energy showed a very slow increase trend during the calm period; After entering a surge period (with the appearance of Kaiser points), both show a significant transition state; During the slow increase period, the overall growth rate of the two slowed down and remained almost silent. (3) On the basis of the analysis of RA-AF values during the deformation and rupture process of diorite, it can be seen that the damage type of diorite is tensile damage by the significant low RA value and high AF value characteristics, which coincides with the actual damage fracture characteristics of the rocks in the sample. (4) During the compaction stage, there are few acoustic emission location points, which correspond to low energy and are mainly distributed at the higher and lower ends of the sample; After entering the elasticity stage, the number of positioning points significantly increases and gradually expands towards the middle; Near Kaiser point, the number of location points and corresponding energy are both in a sharp increase state, and this trend is in good agreement with the changes in the ringing count-time and energy-time curves. (5) The damage time mainly starts at the end of the calm period, and the pattern of change in the damage curve coincides with the localization point and energy evolution. The results of the research can be used as a referential basis for the development of the excavation, protection and other construction plans for the Lingbei TBM section of the Hanjiang-To-Weihe River Qinling tunnel or similar surrounding rock tunnels, as well as for further conducting triaxial unloading tests on diorite.

## 1 Introduction

After the excavation of TBM section of Hanjiang-To-Weihe River Qinling tunnel was completed in the hard rock section with buried depth of more than 1 km, under high geostatic stresses, the material experiences significant unloading, the steel frame of the arch was sunk and the bottom was uplifted, and the phenomenon of rock spalling, falling block and collapse was serious, sometimes leading to TBM stuck and other problems, which seriously affected the normal excavation construction of the tunnel and posed a threat to the stability of the tunnel structure in the later period. Among them, the diorite segment with buried depth of 1808 m is particularly prominent, which offers a theoretic foundation for the formulation of tunnel construction and protection schemes, and there is an urgent need to study how diorite deforms and fractures. Due to external loading, primary cracks within the rock typically close, expand and connect, or generate new cracks. When this phenomenon occurs inside the rock, pulses are usually generated, which are emitted as elastic waves and acoustic emission. AE is an effective method for capturing rock microfracture signals in a timely manner, where a single acoustic emission signal can be interpreted as a transient elastic wave.

Domestic and foreign scholars use theoretical analysis, mechanical experiments, and other methods to extensive research has been conducted on the mechanics of rocks. Chen et al. [1] and Li et al. [2]conducted Brazilian splitting and uniaxial compression tests on various rocks, including sandstone and mudstone. The properties of the deformation and rupture modes of the rocks under various loading conditions were analysed. Su et al. [3] conducted uniaxial compression, triaxial compression, and Brazil splitting tests on red sandstone and analysed its deformation and rupture properties. Niu et al. [4] conducted an in-depth study on the fracture properties of rocks, and concluded that the presence of fractured surfaces has an obvious impact on the peak strength of rocks, but does not have a direct and decisive impact on the residual strength. Huang et al. [5] conducted a uniaxial compression test on chlorite schist, obtained the deformation and failure law of chlorite schist and various characteristic strengths such as closure, initiation, damage and peak strength, and analysed the relationship between these characteristic strengths and peak strength, as well as their anisotropy characteristics. Wang et al. [6] demonstrated the correlation between the initial dilatation load and Young's modulus, Poisson's ratio and porosity through the physical properties and uniaxial compression tests of 32 groups of granite and diorite, and established the prediction equation of the initial dilatation stress of granite and diorite.

Acoustic emission technology is a widely used non-destructive testing technique in the area of rock exploration. Liu et al. [7] conducted uniaxial compression and Brazil splitting tests on coal rock. The deformation and rupture properties of the carbon rock under tensile and compressive loads, the spatio-temporal progression law of AE, and the microscopic rupture mechanism were compared and studied. Wang et al. [8] examined the evolution of fractures in granite under increased fatigue load. The study combined live AE monitoring with a 3D post-test CT scanning technique to determine the fracture evolution characteristics of fractured granite. Wang et al. [9] investigated the evolution of failure law of red sandstone at different cyclic loading and unloading paths based on AE and DIC monitoring technologies. Xie et al. [10] respectively carried out uniaxial compression and Brazilian splitting acoustic emission tests on salt rock, and analysed the AE evolution law in the loading process. Liang et al. [11] investigated the impact of cyclic loading and unloading on the mechanical properties of marillaceous limestone in the after peak phase, and examined the feasibility of acoustic emission amplitude evolution characteristics to assess the precursor of rock yield. Chen et al. [12] conducted uniaxial compression tests on samples of granite, basalt, red sandstone, limestone, and marble. During the test, the internal AE signals of the specimens were monitored, and the combined analysis index of thermal-acoustic sensitivity was proposed. Wang et al. [13] Wang analysed the main parameters of AE signals, including frequency, entropy, energy, event rate, and amplitude, throughout the entire process of rock fracture as preliminary information of rock fracture. Song et al. [14] conducted uniaxial AE tests on limestone with different freeze-thaw cycles, obtained corresponding physical and mechanical parameters, and studied the correlation between AE signals and microcrack activity in freeze-thaw limestone. Wang et al. [15] took deep hard coal as the research object and carried out the sectional high temperature axial static load acoustic emission test with different heating lengths to examine the deformation and rupture properties of the carbon body. Guo et al. [16] prepared cube specimens of varying sizes for a uniaxial compression test. During loading, the acoustic emission and the resistance changes of the samples were monitored, and the impact of the cube size on the fracture and breakage of the gangue-cemented backfill was investigated. Dong et al. [17,18] carried out AE experiments on the instability and fracture of granite, and analysed the phase properties of the two indices of AE energy level frequency distribution and waveform spectrum change in the progress of rock rupture, on the basis of granite expansion micro-crack experiment and RA-AF value distribution, suggestions are put forward to reasonably improve the use of AE characteristic parameters to judge the types of micro-cracks. Feng et al. [19] investigated the displacement variation rule of measuring points on the surface of specimens in the process of uniaxial loading, proposed the formula for calculating the pseudo-variation of the increase in displacement at multiple measuring points on the surface of rocks, and analysed the corresponding relationship between the pseudo-variation of the increase in displacement-variation coefficient and the stress curve. Yang [20] investigated the mechanism of micro-fracture evolution and the properties of AE in rock deformation and rupture processing by hydraulic coupling. Liu et al. [21] conducted acoustic emission tests of limestone under Brazil splitting and uniaxial compression to discuss the B-value characteristics of rock failure AE and the influencing factors of b-value calculation under two loading modes. Zhu et al. [22] carried out acoustic emission tests on samples of sandstone rock under uniaxial loading and compression to explore the AE precursor characterization of rock critical fracture. Qiao et al. [23] took deep granodiorite (564–576 m) as the research object and adopted a microcomputer servo triaxial rock testing machine and AE monitoring system to conduct compression test and AE monitoring test on rock specimen, so as to obtain acoustic emission characteristics during deep granite failure process; Wu et al. [24] based on the digital image correlation (DIC) method, the position of the tip of the crack process zone (FPZ) is calculated. It is found that the opening displacement of the critical crack tip increases in a parabolic direction with the change of heat treatment temperature; Li et al. [25] by using the Hilbert-Huang transform (HHT) method, the analysis of AE waveform properties provides detailed structural characteristics of the coal and rock mass in relation to damage at different loading levels.

Obviously, the scholars mentioned above have made significant successes in researching the mechanical properties and AE properties of various rocks under various loading conditions. However, research on the macro-mechanical characteristics of rock and the micro-fracture characteristics of AE are relatively independent of each other, and the mutual verification analysis of the two research results is rarely carried out, so as to uniformly reveal the deformation and fracture mechanical of rock. Therefore, uniaxial compression and AE tests were conducted on diorite rock specimens at a burial depth of 1808 m. While systematically studying the macroscopic mechanical characterization of diorite uniaxial compression and the microscopic damage and fracture characteristics of acoustic emission, a comprehensive confirmatory analysis was conducted to reveal the deformation characteristics, failure mode and damage and fracture mechanism of diorite uniaxial compression. The investigation outcomes can serve as a reference for the construction scheme of tunneling and protection of Hanjiang-To-Weihe River Qinling tunnel and similar surrounding rocks, and the further triaxial unloading test of diorite.

## 2 Experimental condition

### 2.1 Specimen preparation

**Fig. 1:**
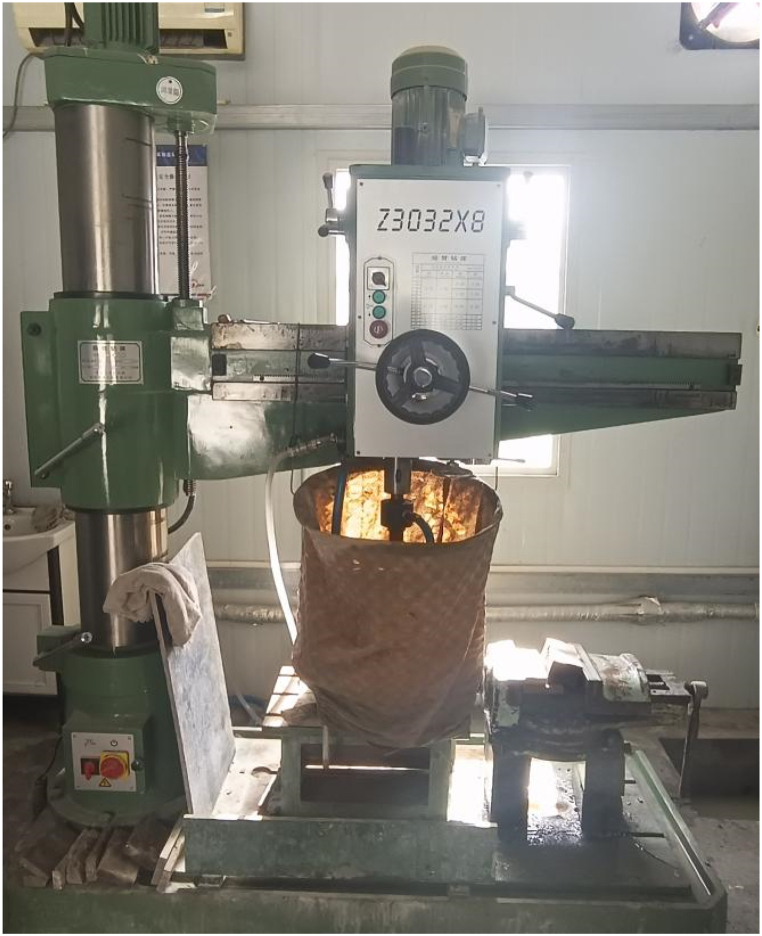
Z3032X8 radial drilling machine.

**Fig. 2:**
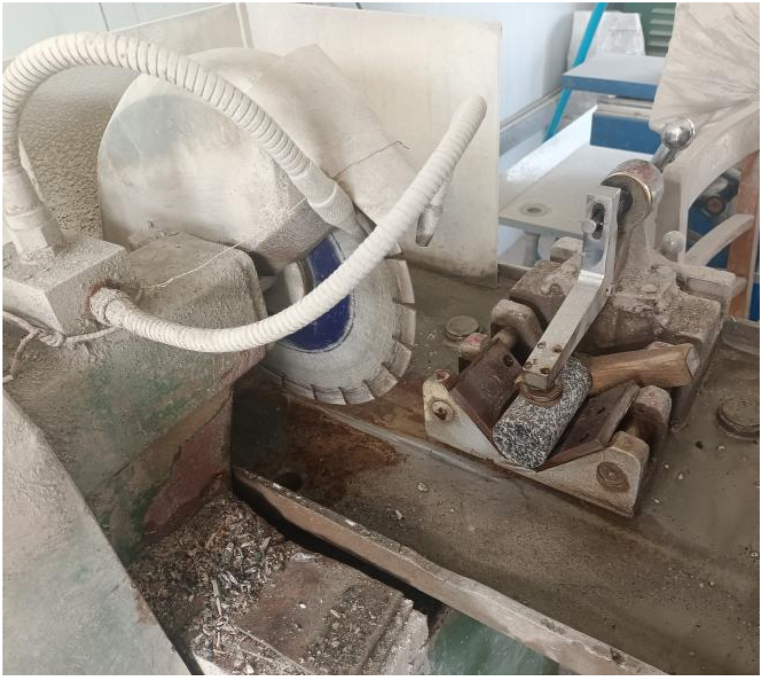
SPQJ-300/338 microtome.

**Fig. 3:**
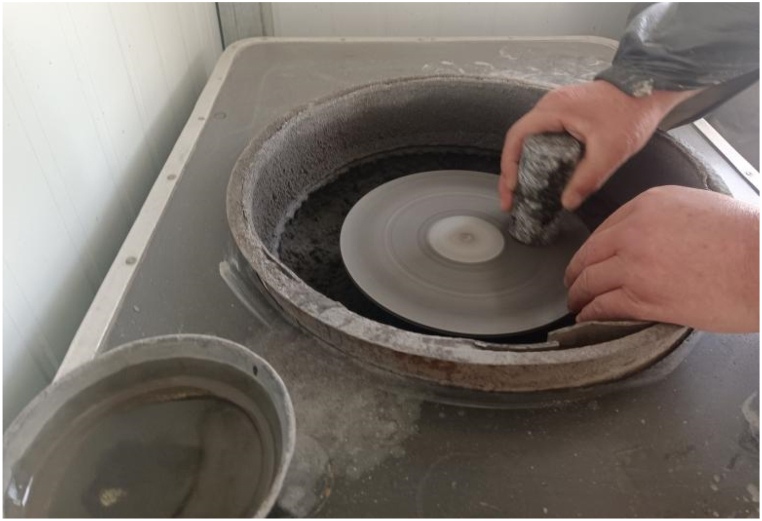
250 single disc grinding machine.

**Fig. 4:**
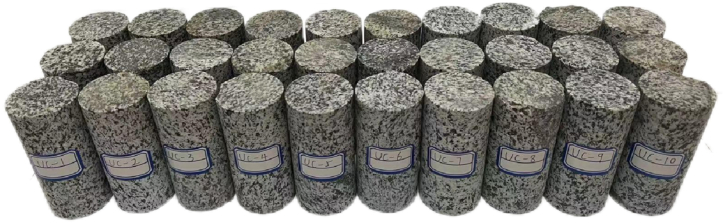
Diorite samples.

The original diorite specimens were obtained from the Lingbei engineering section of the TBM construction section of the Qinling Tunnel, which is buried at a depth of 1808 m. Sample preparation is carried out strictly according to GB/T 23561.7–2009 "Method for Determination of physical and Mechanical Properties of Coal and Rock". First, Z3032X8 radial drilling machine was used to core the test block (Fig. 1), and then SPQJ-300/338 microtome was used to cut it (Fig. 2). Finally, it was polished by 250 single disc grinding machine (Fig. 3) and manufactured into standard diorite sample sizes of Φ50 mm × 100 mm (Fig. 4).

### 2.2 Test unit

Test with 2 sets of loading control system and AE monitoring system.

(1) Load equipment and conditions

The loading equipment is MTS-2000 microcomputer controlled electro-hydraulic servo universal testing machine. In accordance with the method of loading proposed by the ISRM [26], the axial displacement control is used, the test load rate is 0.005 mm/s, from the initial loading until the destruction of the specimen, the final full stress-strain curve is obtained.

(2) Acoustic emission system and monitoring preparation

①System introduction. The acoustic emission device adopts DS5-16B acoustic emission instrument of Beijing Softisland Times Technology Co., LTD. The acoustic emission system has 16-bit A/D conversion, the pass rate of continuous data is 131 MB/S, and the pass rate of waveform data is 96 MB/S. The sampling speed varies according to the channels used. 4 channels are used, and 8 channels are used for each 10 M channel. 16 channels are used for each channel 6 M or 5 M, and 3 M or 2.5 M for each channel are used for multi-channel synchronous data acquisition. A 4 TB hard disk is used for synchronous acquisition of 16-channel acoustic emission signals with 3 MHz sample rate. Waveform data can be stored continuously for 11.6 h, ensuring no data loss during the period, with good overall stability and high sensitivity. It can support acoustic emission signal detection of multiple channels. This test is equipped with 8 acoustic emission signal acquisition probes. According to the properties of the diorite samples, the AE sampling threshold is set at 40 dB, the pre-amplification gain is set at 40 dB and the sample frequency is set at 3 MHz.

②The basic parameters of the specimen were measured. At present, the spatial positioning methods for acoustic emission events of rocks are basically founded on the time differential of P wave or S wave generated by each AE event to arrive at different sensors and the corresponding wave velocity to determine the position coordinates [27]. Therefore, to enhance spatial positioning accuracy of diorite AE events and reduce survey error, the geometric shape of each sample was established prior to the experiment by means of a vernier caliper, and the position coordinates of sensor (probe) were determined. The measurement results of acoustic wave (P wave) velocity calibrated by NM-4B nonmetallic ultrasonic detection analyzer are listed in Table 1, where D is the specimen diameter. H is the specimen height; ρ means rock sample density; σ_m_ stands for peak intensity; E is Young's modulus; ε_1m_ indicates peak axial strain.

**Table 1:** Basic parameters and uniaxial compression test results of diorite samples.

Specimen number	D /mm	H /mm	Ρ /g/cm^3^	σ_m_ /MPa	ε_m_ /%	E /GPa	Wave velocity /km/s
UC-1	49.9	100.1	2.56	90.00	0.74	18.73	2632
UC-2	50.1	100.2	2.56	73.50	0.81	11.42	3165
UC-3	50.0	100.3	2.55	102.42	0.84	16.52	2778
UC-4	49.8	100.0	2.65	77.97	0.80	13.79	2500
UC-5	50.1	100.5	2.49	93.06	0.92	11.08	2358
UC-6	50.0	100.4	2.56	71.55	0.90	12.61	2525
Mean value	49.98	100.25	2.56	84.75	0.84	14.03	2660
Variation coefficient%	0.21	0.17	1.82	13.24	7.31	19.72	9.77

**Fig. 5:**
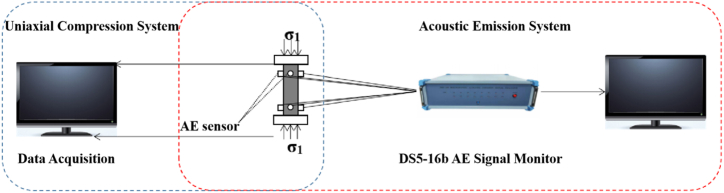
Diagram of diorite uniaxial compression acoustic emission system.

**Fig. 6:**
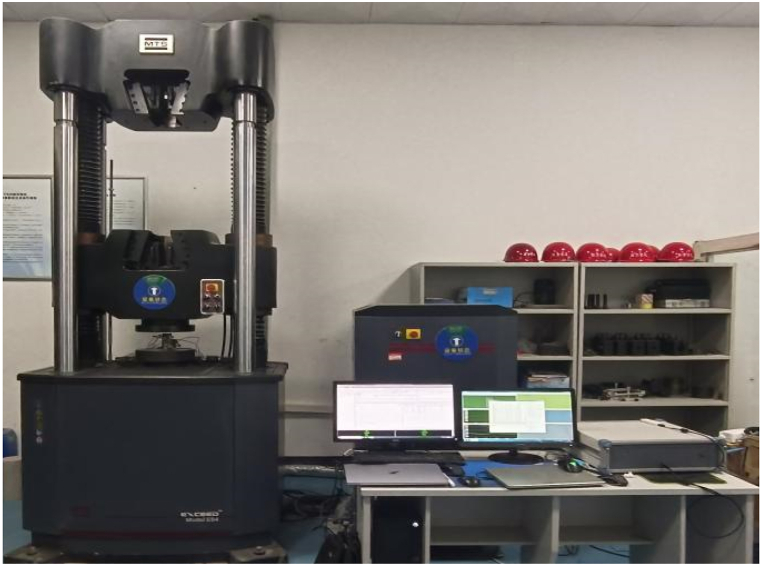
Physical picture of diorite uniaxial compression acoustic emission system.

③Probe (sensor) layout. Since the rock sample is a cylindrical standard specimen, to pinpoint exactly where the sound originated, probes are arranged in a double-layer surround monitoring network [28]. The eight probe positions are arranged in a spatial right-angled coordinate system with the centre of the bottom surface of the specimen as the coordinate origin, with the following coordinates: (25, 0, 20), (0, 25, 20), (−25, 0, 20), (0, −25, 20), (25, 0, 80), (0, 25, 80), (−25, 0, 80), (0, −25, 80), the probes 1–4 and 5–8 are arranged respectively under the specimen and on the upper two planes. 302 glue is used to paste between the probe and the specimen, and then connected with the preamplifier, acoustic emission instrument, computer, etc., to form the AE monitoring system (Fig. 5). The current image is displayed in Fig. 6.

During the experiment, the computer records stress and strain data in real-time while the sample undergoes uniaxial loading and failure. The resulting test curve is then plotted. The AE system monitors the AE parameter characteristics of the sample's deformation and failure process. During the experiment, it is ensured that the loading and acoustic emission monitoring processes are synchronized in time, and AE parameters are read and collected at equal time intervals.

## 3 Experimental results and analyses

### 3.1 Mechanical properties of diorite in uniaxial compression

**Fig. 7:**
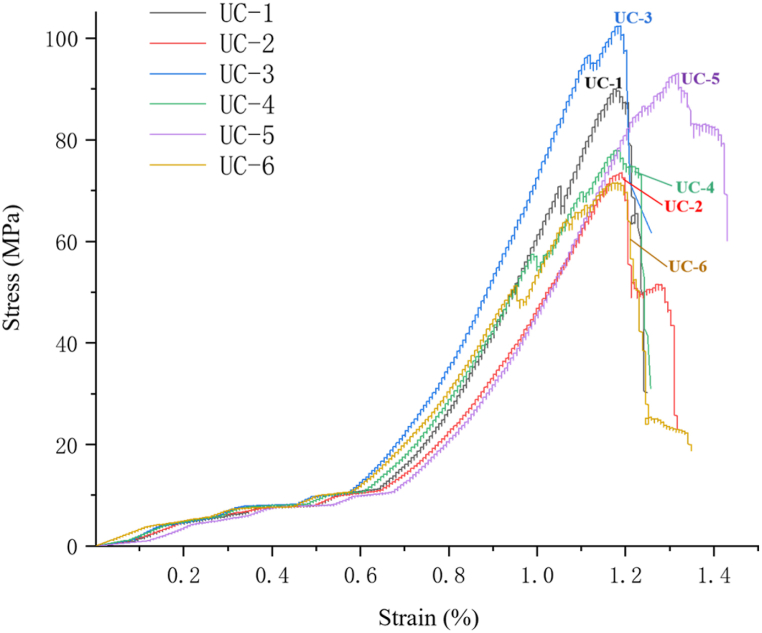
Stress-strain curve.

In this experiment, 10 diorite specimens were chosen for uniaxial compression testing and the test data from six rock samples with representative rock sample conditions and test results were selected for further analysis. Fig. 7 displays the stress-strain curve. The figure clearly shows that each specimen undergoes four stages during the uniaxial compression process: initial compaction, elasticity, yield, and failure. These stages relate to the deformation properties of the specimen. The deformations of the samples at the initial compaction stage are evident, indicating that there are many primary microcracks or defects in the diorite. In most specimens, the stress increases faster than the strain in pre-peak elasticity stage, and the curve rises smoothly. During the yield stage, there is a fluctuation in stress that rises in a stepped pattern. Following this stage, there is a rapid drop in stress during the post-peak failure stage, which is then followed by a further decrease. It is obvious that the deformation characteristics of diorite are generally stable.

**Fig. 8:**
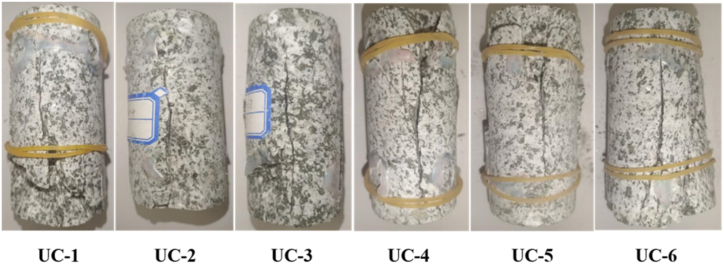
Failure state of diorite.

In terms of strength characteristics, Fig. 7 in combination with Table 1 shows that the uniaxial compressive strength of 6 diorite specimens ranges from 71.55 to 102.42 MPa, with an average of 84.75 MPa, and the variation coefficient (ratio of standard deviation to mean) is 13.24 %, indicating that uniaxial compressive strength has a high dispersion. In terms of failure characteristics, Fig. 8 shows that the specimen failed due to a crack along its central axis, showing a splitting failure mode. In addition, from the perspective of brittleness ductility, referring to the engineering classification of axial peak strain (ε_m_) based on uniaxial compression, that is, brittle failure is when ε_m_ < 1 %, brittle failure is when ε_m_∈ (1 %, 5 %), and ductile failure is when ε_m_ > 5 % [29], the peak axial strain of diorite in this test is between 1 % and 1.4 % (Fig. 7). The brittle ductility failure is the main failure mechanism, so the brittle ductility splitting failure is the overall failure mechanism. It is clear that how to enhance the resistance of the overburden (tensile failure capability) is a key link in controlling the stability of the overburden after excavation. The currently synthesis of various research approaches generally based on the step-by-step joint support concept for the enhancement of perimeter rock resistance in the field [[30], [31], [32], [33]].

### 3.2 Acoustic emission characteristics of specimens under uniaxial compression

AE ringing count, energy, RA-AF and location point parameters in the uniaxial compression test of diorite were used to study the properties of AE parameters in the deformation and fracture processing of rock samples. The test data of typical specimens UC-1∼UC-6 were taken as examples for analysis.

#### 3.2.1 Analysis on the evolution characteristics of energy, stress and ringing count

**Fig. 9:**
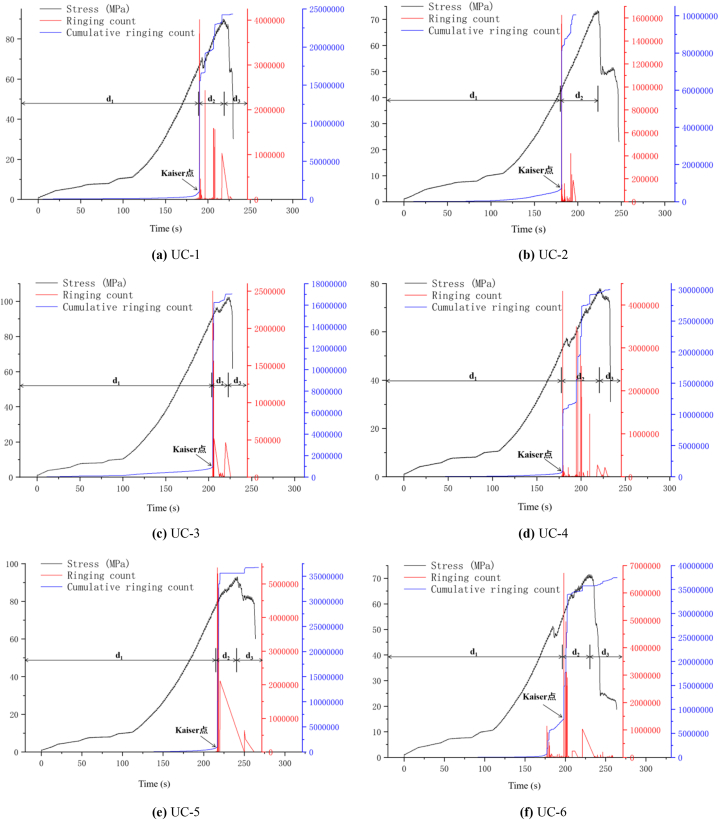
AE ringing count changes with stress and time (a)UC-1, (b)UC-2, (c)UC-3, (d)UC-4, (e)UC-5, (f)UC-6.

**Fig. 10:**
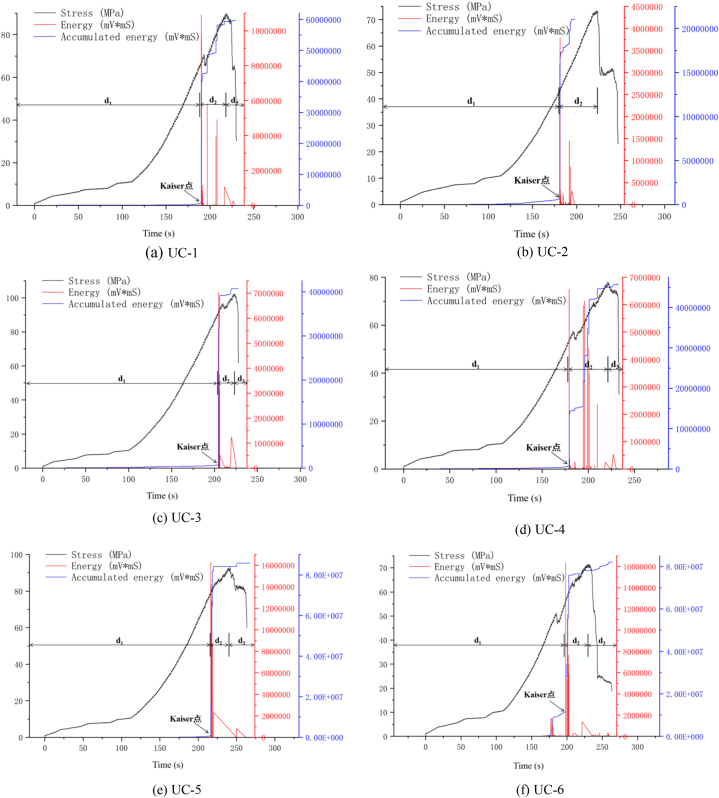
AE energy changes with stress and time (a)UC-1, (b)UC-2, (c)UC-3, (d)UC-4, (e)UC-5, (f)UC-6.

Fig. 9(a)∼(f) and Fig. 10(a)∼(f) show the change curves of stress, energy and ringing count over time during the entire uniaxial compression test of uniaxial compression test of typical diorite specimens. Since the overall evolution process of ringing count and energy can be classified into three stages: calm, surge and slow increase period, and the changing trends of the two phases are almost the same, the evolving features of the ringing count are mainly analysed as follows. For the convenience of observation, d_1_ represents the calm period, d_2_ represents the surge period, and d_3_ represents the slow increase period in Fig. 9∼10.

(1) Calm period. The ringing count and cumulative ringing count (Fig. 9(a)∼(f)) both show a slow growth trend, and there are only a few AE events. The analysis shows that at this phase the diorite samples are in the process of asymptotic compaction and elastic compaction of the initial defects and microcracks, and the internal structure of the rock samples tends to be complete as a whole, and the probability of new damage and fracture is small, which does not have the conditions for obvious AE activities.
(2) Surge period. As shown in Fig. 9(a)∼(f), The ringing count (ringing count-time curve) changes significantly and the cumulative ringing count (cumulative ringing count-time curve) shows a large transition state when the rock sample is loaded to the end of the elastic phase before yielding (stress-time curve). It is easy to judge, obviously at this time, there is obviously a new damage fracture in the rock sample. According to Kaiser effect, when the stress on the rock sample exceeds the historical maximum stress, large number of AE phenomena generated [34]. Combined with the method of determining Kaiser points by cumulative ringing count-time curve or cumulative energy-time curve [27], the large jump point of cumulative ringing count, namely the steep increase point, is Kaiser point. From Fig. 9(a)∼(f) and Fig. 10(a)∼(f) it is easy to see that the Kaiser point of UC-1 is 189.80 s and the homologous stress is 57.75 MPa. The kaiser point of UC-2 is at 180.55 s, and the homologous stress is 42.74 MPa. The kaiser point of UC-3 is at 204.58 s, and the homologous stress is 91.48 MPa. The Kaiser point of UC-4 is at 179.04 s, and the homologous stress is 54.06 MPa. The Kaiser point of UC-5 is at 216.56 s, and thehomologous stress is 70.95 MPa. The Kaiser point of UC-6 is at 197.18 s and the homologous stress is 55.46 MPa. Obviously, the stress at the steep point is the largest load that the rock sample has been subjected to in its history. After passing the steep rise point, the value of the number of rings showed a clear downward trend and the increase rate of the cumulative ringing count decreased significantly. According to the analysis, after reaching the plastic yield stage, the microcracks and microdefects in the sample expand rapidly and a high number of new fractures are generated. At the same time, they are further merged and connected, resulting in macroscopic fracture surface and extremely active AE activities.
(3) Slow increase period. After the steep increase period (after the peak stress), the increase rate of the cumulative ringing count curve is generally slower and almost horizontal (Fig. 9(d)–Fig. 9 (e), Fig. 9 (f)) except for some cases with a small increase (Fig. 9 (a) and 9 (c)). Although the probe of the UC-2 sample fell off during the test, no new data was collected after the steep increase period, but it did not affect the result analysis. The analysis shows that the specimen slides along the macroscopic fracture plane as the rock achieves maximum strength. Although the stress drops rapidly in the process of continuous increase of strain, there are still a few new cracks.

In summary, in the uniaxial compression test of diorite, at the final stage of compression and elasticity, the AE activity is in a calm period and the cumulative ringing count time and cumulative energy time curves show a weak increasing trend; After the Kaiser point appears, microcracks and micro defects rapidly expand, converge, and coalesce, resulting in macroscopic failure of the sample. AE activity is particularly intense at this phase; During the post-peak stage of the stress-strain curve, only a limited number of new cracks are formed and the AE activity is in a quiescent state.

#### 3.2.2 Analysis of RA-AF value

**Fig. 11:**
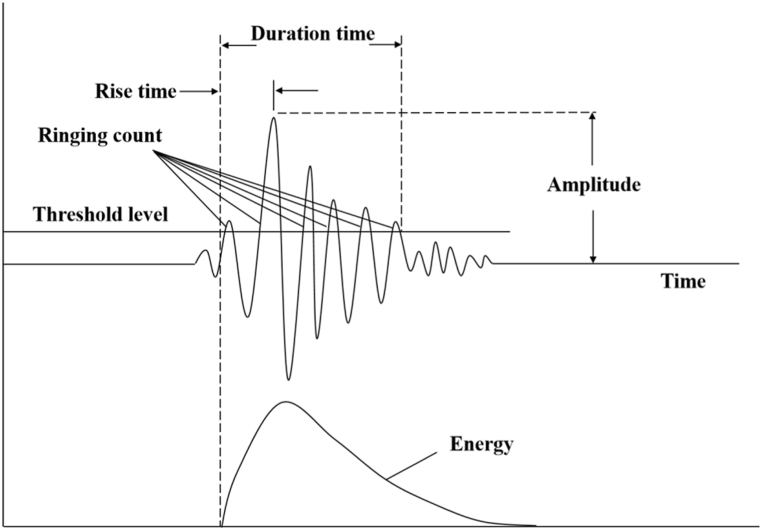
Schematic diagram of AE parameter [40].

In the study of acoustic emission characteristics, RA value (rise time to amplitude ratio, ms*V^−1^, Fig. 11 [40], the same below) and AF value (count to duration ratio, unit kHz) are commonly used to analyze rock fracture modes. Shear failure is indicated by a high RA and low AF, while tensile failure is indicated by a low RA and high AF [[35], [36], [37], [38], [39]]. The RA value's magnitude indicates the signal's abruptness to some degree. The smaller RA value is, the narrower the pulse is, the more sudden the signal characteristics of the waveform are, and usually the higher the frequency is, which is exactly in line with the characteristics of the transient signal generated by the rock fragmentation failure. On the contrary, the larger the RA value, the wider the pulse, the more sustained the signal characteristics of the waveform, and generally the lower the frequency, which is exactly in line with the delayed signal characteristics caused by rock shear failure. However, the waveform range described by RA value is only for the rising period, and the burst or width of the pulse cannot be completely determined, the AF value represents, to some extent, the frequency properties of the entire pulse, which can make up for the deficiency of RA value. Therefore, the distribution map of RA-AF can be used for more accurate measurement of waveform properties.

**Fig. 12:**
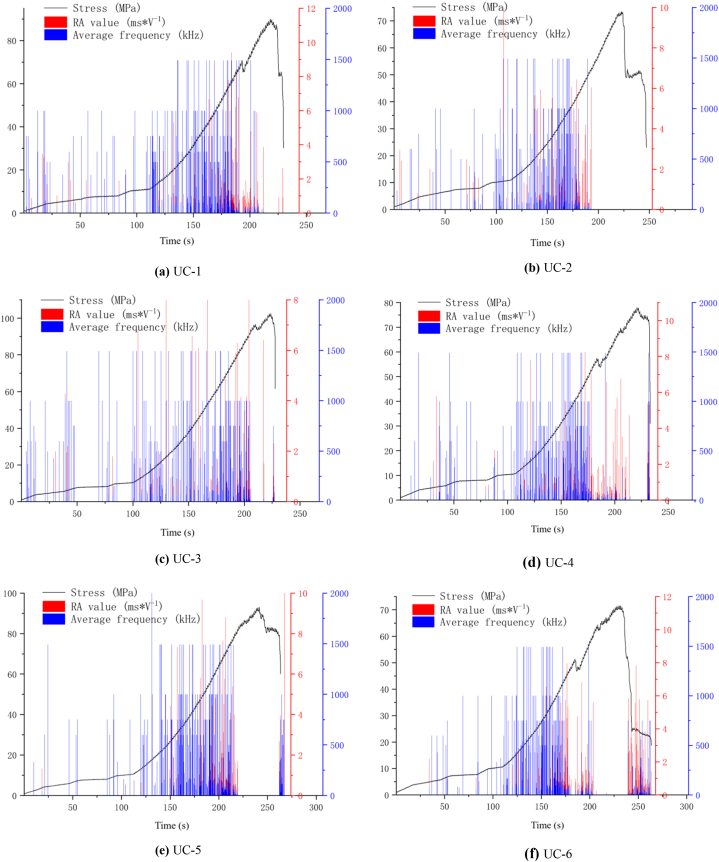
Distribution of stress and AE RA and AF values in diorite (a)UC-1, (b)UC-2, (c)UC-3, (d)UC-4, (e)UC-5, (f)UC-6.

Fig. 12(a)∼(f) illustrates the variation in RA, AF and stress of diorite samples UC-1∼UC-6 over time. The variation of RA and AF values can be classified roughly into three stages, bounded by the compaction-elastic transition point and the Kaiser point of the stress-time curve. In the first stage (compaction stage), AF value is characterized by high value and sparse distribution. While RA value is high, but the number is very small, and the bar graphs of both do not show a stable growth or decline rule. Based on the analysis, the primary cause of this phenomenon is the initial load, there are more unevenly distributed micro-cracks or defects inside the rock, and certain fracture damage occurs when the rock is closed, so that the tensile crack is dominant. However, at this stage, the slip between the lattice is very little, so the shear crack is not obvious. As the axial stress gradually increases, the rock enters the second stage, which spans from the elastic stage to the Kaiser point. The dispersion density of RA and AF values increased significantly, their values fluctuated over time. Generally speaking, they could be divided into two situations: One is shown in Fig. 12 (a) UC-1, Fig. 12 (d) UC-4, and Fig. 12 (f) UC-6, the high AF value is mostly present, while the high RA value sometimes appears. The analysis indicates that the primary cause of this phenomenon is the increase in strain energy during the elastic compaction process of the rock sample, which creates the conditions for the initiation and propagation of internal tensile cracks. The inhibition of tensile crack propagation creates conditions for shear slip at the end, which can also be inferred from the final failure mode of the sample (UC-1, UC-4 and UC-6 in Fig. 8), that is, the vertical tensile crack is dominant, but the end has obvious transverse fracture. The other is shown in Fig. 12(b)–Fig. 12 (c), and Fig. 12 (e), where the high AF value is obviously most, and the high RA value rarely appears. The analysis suggests that this is because the two ends of the rock sample are smooth, and the friction at the end has little effect on the middle section of the sample. Starting from Kaiser point and entering the third stage, although AF value is still floating at a higher level, RA value presents a sharp increase trend, and the distribution density increases as the RA value approaches the yield point (except Fig. 12 (a) UC-1 and Fig. 12 (f) UC-6, because UC-1, UC-6 macro-tensile cracks have formed before Kaiser point), while the distribution density of AF value presents a shrinking trend. At this phase, tensile fractures in the rock specimen begin to coalesce and form macro-scale initial cracks that are oriented in the direction of loading. Because of the increase in internal failure and the asymmetry and irregularity of the cracks, frictional slip is easy to occur between the interfaces of micro-cracks, that is, shear cracks occur in a brief period, leading to the raise of RA value. Generally, shear cracks develop after the formation of macro-scale tensile fractures, so the AF value is usually reduced close to the failure point.

To sum up, during the deformation and rupture process of diorite, the low RA and high AF characteristics are more significant, and the RA-AF values of the six groups of rock samples UC-1∼UC-6 are basically consistent. Therefore, it can be summarize that the fracture mode of the diorite is tensile failure, which is in accordance with the actual fracture of the rock samples shown in Fig. 8. The failure mode of diorite can be judged by using the variation trend of RA-AF value.

#### 3.2.3 Evolution characteristics of AE location point and energy

**Fig. 13:**
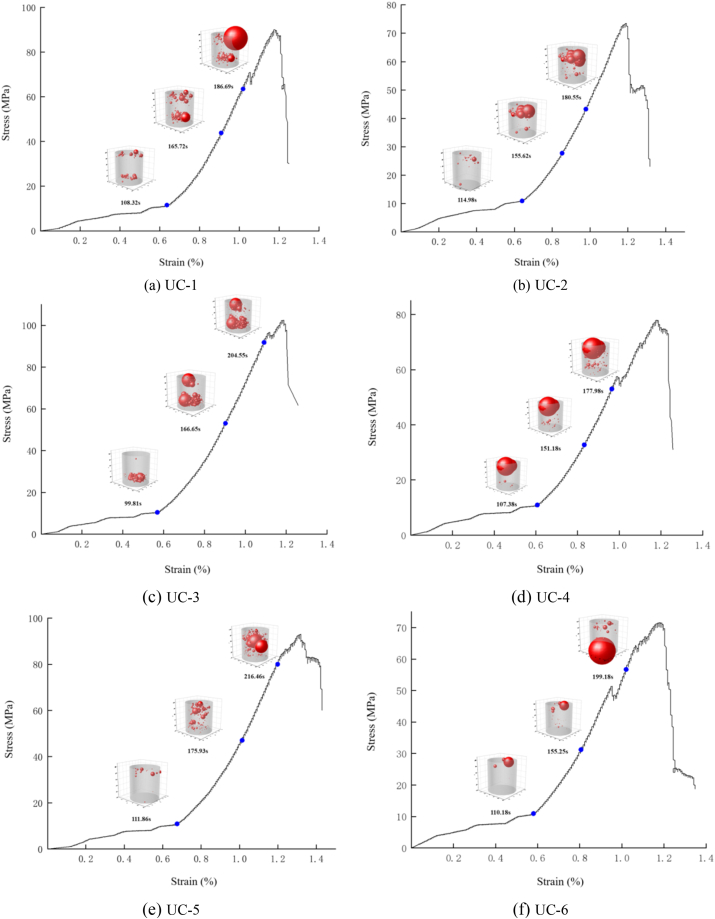
AE location point and energy evolution of diorite at different stages (a)UC-1, (b)UC-2, (c)UC-3, (d)UC-4, (e)UC-5, (f)UC-6.

Acoustic emission positioning can monitor and reproduce the spatial distribution of rock sample fracture in real time, and the amount of energy released at the location point can reflect the intensity of rock sample failure. Fig. 13(a)∼(f) display the AE positioning during uniaxial compression of typical specimens UC-1∼UC-6, the energy evolution progress with time and the corresponding relationship between the stress-strain curve (the quantity of red pellets in the figure represents the number of location points. Its volume represents energy). However, due to the limitation of acoustic emission instrument monitoring capability, that is, after intense acoustic emission activities, the take-off point of acoustic emission waveform is often confused (i.e. the waveform take-off point is unclear, resulting in the failure of the accepted location signal [41], so that the location point signal cannot be accepted. For this test, The above phenomena occurred immediately after Kaiser point, and no location point signal was detected. Therefore, the scatter map of location point-energy space in Fig. 13(a)∼(f) was drawn from the actual received AE signals.

Fig. 13 shows that the acoustic emission location points during the uniaxial compaction stage are generally limited, indicating low energy. These points are mainly distributed at the higher and lower ends of the rock specimens. Analysis suggests that the damage to the rock specimens during the compression process is mainly due to unloading damage or external force disturbance during the collection and processing process, resulting in more microcracks or micro defects at the end of the rock sample than in the middle. Fig. 13 (d), except for UC-4, where the rock blocks at the end of UC-4 fly off during the initial compression stage. Simultaneously, the AE probe dislodged, causing the UC-4 to have a high energy localization point during the compaction stage. After reaching the elastic stage, the quantity of positioning points significantly rises, with a small rise in energy, and it still develops mainly at the two ends of the rock specimen, but there is a tendency to gradually expand towards the centre. The reason is that although the rock sample has completed compaction, there are still more weak surfaces at both ends compared to the middle. Under axial load, for rock samples with friction on the end face and obvious end effect, such as UC-1 in Fig. 13 (a), the end is in a three-dimensional compression state, The fracture damage of rock samples is caused by shear slip of their internal weak planes; For rock samples with smooth ends and insignificant end effects, such as UC-5 in Fig. 13 (e), the internal deformation damage is caused by microscopic tensile stress (Poisson effect). Near the Kaiser point, the number and corresponding energy of the positioning points are in a sharp increase state, with continuous cracking sound and the maximum cracking sound accompanied by the maximum energy. The observed tendency of variation is in accordance with the pattern of variation of the ringing count time (Fig. 9) and energy time (Fig. 10). Although the positioning point signal cannot be obtained after the Kaiser point, the mechanical and acoustic emission parameter characteristics mentioned above can be used to clearly distinguish the deformation and fracture characteristics of the rock specimen by analysing the mechanical characteristics of the rock specimen.

#### 3.2.4 Study of uniaxial compression damage mechanism based on acoustic emission

Acoustic emission parameters can effectively characterise changes of material characteristics, and have a good correspondence with the internal fine lattice displacement and fracture evolution properties of material deformation, and can be used to measure differences in the fracture state of the rock using the cumulative ringing count rate as a property parameter [42,43], the variable D, which represents damage, is defined as:

(3.1) $$D=\frac{A_{d}}{A}$$

The formula defines A_d_ as the fault area when the rock specimen is broken under pressure, and A as the approximate fault area when the rock specimen is undamaged prior to loading.

If we set the cumulative ringing count that corresponds to the entire destruction of the rock sample to V_0_, and the ringing count that corresponds to the destruction of its unit area to V_w_:

(3.2) $$V_{w}=\frac{V_{0}}{A}$$

During loading, the rock specimen's fracture area is destroyed continuously till the cumulative ringing count rate corresponding to A_d_ is equal to V_d_:

(3.3) $$V_{d}=V_{w}A_{d}=\frac{V_{0}}{A}A_{d}$$

In summary, the relationship between damage variable D and ringing count rate is:

(3.4) $$D=\frac{V_{d}}{V_{0}}$$

**Fig. 14:**
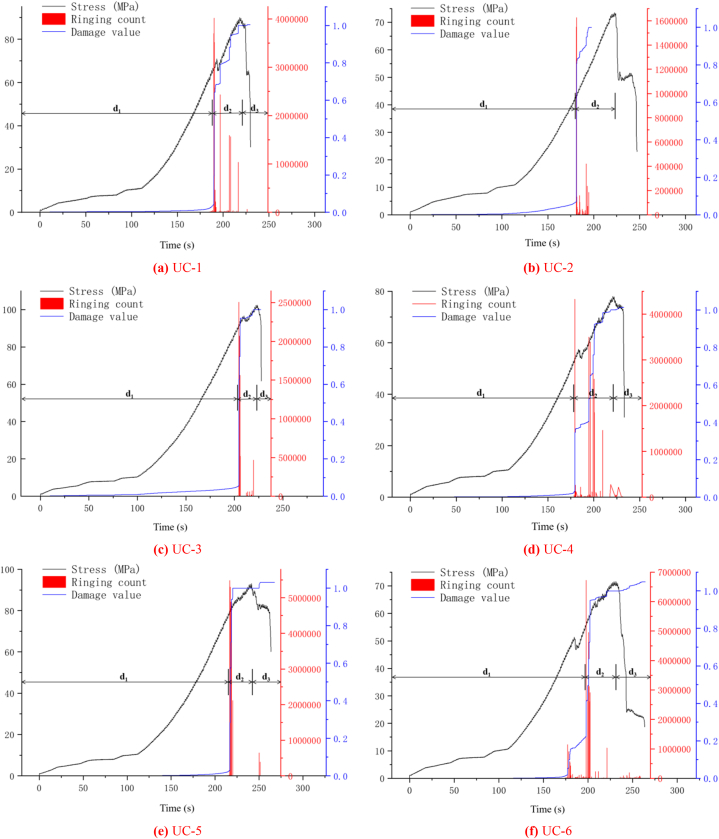
Damage evolution of diorite under uniaxial compression test (a)UC-1, (b)UC-2, (c)UC-3, (d)UC-4, (e)UC-5, (f)UC-6.

Using the above damage value calculation method, the entire process damage evolution curve of diorite uniaxial compression test is drawn, as illustrated in Fig. 14(a)∼(f). Considering the crack value time and ringing count time evolution characteristics of different rock samples in the diagram, the crack evolution stage can be classified into three stages: damage calm period, damage surge period, and damage slow increase period. The specific details are as follows.

(1) Damage calm period. As can be seen from Fig. 14(a)∼(f), almost no new micro-fractures and defects appear or grow little in diorite from the initial loading stage, and the damage value-time curve does not show a significant growth trend until the end of the compaction stage, indicating that the new micro-fractures and defects in diorite do not gradually increase after the compaction stage. However, the damage degree is also very low (except for UC-6 specimen (Fig. 14 (f)), the damage value increases significantly near the steep increase point, which is due to the reason for this is the occurrence of larger energy loci at the end of the rock samples at the end of the compacting phase of UC-6 (Fig. 13 (f)).
(2) Damage surge period. Compared with the damage calm period, the growth rate of the damage value-time curve at this stage was significantly increased (Fig. 14(a)∼(f)). Based on the analysis of the ringing count above, it is evident that AE events occur frequently within the rock specimen at this time point, and the concentration of high energy fixed points is evident. It is evident that the cracks within the rock specimen are developing at a high rate at this time and are almost fully developed (Fig. 13(a)∼(f)).
(3) Damage slow increase period, the compressive strength of diorite has reached the critical value at present, the value and quantity of the AE ringing count showed a decrease, and the damage value-time curve showed a slow increase at this phase, which shows that the cracks in the specimen mass have been thoroughly through and formed macro cracks after the diorite samples have reached the uniaxial compression peak. However, as the loading continues, the rock blocks around the macro-cracks of the rock sample begin to slip, and new cracks will occur until the main body of the rock specimen completely collapses, and the internal defect reaches its peak at this time.

To sum up, the process of damage evolution in diorite can be classified into three phases: damage calm, surge and slow increase period. During the calm period, minimal damage occurs within the rock specimen; During the surge period, the rock specimen essentially completes the fusion of the cracks; During the period of slow increase, the rock sample's failure value peaks and shows macro-penetration cracks.

## 4 Conclusions

Drawing from the experimental investigation of deep diorite's mechanical properties under uniaxial compression and its acoustic emission characteristics, the following conclusions are obtained:

(1) During uniaxial compression, deep diorite goes through four stages: initial compaction, elasticity, yield, and failure. The curve is noticeably smoother at the initial compaction step, which suggests that it contains a large number of original microcracks or other imperfections; Uniaxial compressive strength ranges from 71.55 to 102.42 MPa, with an average of 84.75 MPa; The axial peak strain ranges from 1 % to 1.4 %, and the overall behavior is characterized by brittle ductile splitting failure.
(2) The process of AE ringing count and energy evolution of diorite can generally be divided into three stages: a quiet period, a surge period and a slow increase period. During the calm period, there are very few AE events, and the cumulative ringing count and energy exhibit a gradual increase; After entering the surge period, the Kaiser point appeared, and the cumulative ringing counts and energy showed a large jump, and the Kaiser point appeared in 179.04s–216.56s, corresponding to the stresses of 42.74 MPã91.48 MPa; During the retardation period (after peak stress), the cumulative ringing counts and energy growth rate slowed down in general and were almost horizontal, except for a few isolated cases of small increases.
(3) Through the analysis of the AE RA-AF values, it is noted that the deformation and fracturing of the diorite is characterised by low RA and high AF, and it can be known that the damage type of the diorite is tensile damage, which matches with the characteristics of the actual damage fracture of the rock samples, and the results demonstrate the feasibility of using RA-AF values to determine the damage mode of diorite.
(4) During the uniaxial compression phase, the quantity of AE location points is generally low, as is the corresponding energy, which is mainly distributed at the higher and lower ends of the rock specimen. After entering the elasticity stage, the number of positioning points significantly rises, with a small rise in energy and a gradual expansion towards the centre. Near the Kaiser point, the number of positioning points and the corresponding energy are in a sharp increase state, with continuous cracking sound and the maximum cracking sound accompanied by the maximum energy occurring, this trend is consistent with the change patterns of ringing count time and energy time.
(5) The process of damage evolvement of diorite can be classified into three phases: damage calm, damage surge and damage slow increase period. During the calm period, there is almost no damage inside the rock specimen. During the surge period, the fractures in the rock specimen merge and connect. During the slow increase period, as macroscopic cracks appear, the failure value of the rock specimen peaks.

## Data availability statement

Data will be made available on request.

## Ethics declarations

All participants/patients (or their proxies/legal guardians) provided informed consent to participate in the study.

Review and/or approval by an ethics committee was not needed for this study because the study focused primarily on rock mechanical properties and acoustic emission characteristics and did not involve human or animal intervention, observation or data collection, there were no potential implications for personal privacy or animal welfare, and no ethics committee review and approval was required.

## CRediT authorship contribution statement

**Kai-De Liu:** Writing – review & editing, Writing – original draft, Software, Funding acquisition, Formal analysis, Data curation, Conceptualization. **Yu Zhou:** Writing – review & editing, Writing – original draft, Software, Project administration, Formal analysis, Data curation, Conceptualization. **Xiao-Ping Zhang:** Funding acquisition, Conceptualization. **Shao-Jun Fu:** Supervision, Conceptualization. **Quan-Sheng Liu:** Conceptualization. **Peng Dong:** Investigation, Conceptualization. **Kai-Wen Yao:** Software, Project administration, Investigation. **Ding-Bo Wang:** Software, Project administration, Investigation.

## Declaration of competing interest

The authors declare that they have no known competing financial interests or personal relationships that could have appeared to influence the work reported in this paper.
